# Associations Between Depressive Symptoms, Appetite and Food Intake Among Hospitalized Older Adults

**DOI:** 10.1093/geroni/igaa057.3376

**Published:** 2020-12-16

**Authors:** Amos Rogozinski, Anna Zisberg

**Affiliations:** University of Haifa, Haifa, Israel, United States

## Abstract

Inadequate food intake is common among hospitalized older adults and is linked to negative hospitalization outcomes, including functional decline and mortality. Depression is a well-established risk factor in inadequate food intake in the community but its role in food consumption during hospitalization is poorly studied. To examine the associations between depressive symptoms, appetite and the quantity of food consumed by older inpatients, we conducted a secondary data analysis of 724 hospitalized adults aged 69 to 95 using a prospective cohort dataset: Hospitalization Process Effects on Functional Outcomes and Recovery. Depression was evaluated with Tucker’s Short Zung Instrument at time of admission. Food intake and appetite were examined daily for three consecutive days, using self-reports of food consumed at breakfast, lunch and supper, based on the nDay Express Questionnaire. Approximately 40% of respondents reported eating half or less than half of each meal. The risk of depression was prevalent among a third of respondents, 54% of whom were identified at high risk of depression. The association between depression and inadequate food intake was found to be negative [F(2,716)=9.00 ,p=0.000 ,η2=0.025]. Low appetite was significantly linked to reduced food consumption [β=-0.39, t=-12.06, p=0.000] and made a considerable contribution to the explained variance of food consumed [F-change (1,717)=145.41 , p=0.000]. Finally, decreased appetite partially mediated the association between depressive symptoms and food intake during hospitalization (B=-0.001, UCI=-0.001; LCI=-0.002). These findings contribute to the understanding of inadequate food intake during hospitalization and indicate the importance of screening for depression among hospitalized older adults.

